# Cancer of the Ileum

**Published:** 1882-09

**Authors:** 


### CANCER OF THE ILEUM.

Primitive cancer of the intestines is much less frequent than cancer of the stomach; the most frequently affected intestines are the rectum, the cœcum and the ileum. Many authors consider the cancer of the small intestines, and especially that of the duodenum, as a secondary form. Dr. De Castel made a good many observations on this subject, and he points to the fact that most cancers develop on the termination of the different canals in the human economy. The portion of the ileum next to the ileo-cœcal valve is often the seat of epithelioma; this part is also essentially the seat of cancer of the ileum, and there are cases where there exists a circumscribed cancer of the valve. The most usual alteration is found above the valve, and then the glands are transformed to white hard masses, with some colloid corpuscles. The induration of the glands impedes the action of this organ, and hence the continued constipation. There are further voluminous ramifications of the mucous membrane which will diminish the cavity of the ileum, and there will be at last a complete obstruction of the canal, so that it is often impossible to inject any water through the canal in autopsy. The cancer will be confined either to the termination of the ileum, or it will attack the neighboring parts, especially the cœcum, which is transformed to a rigid canal devoid of any elasticity. In the latter cases it is difficult to distinguish the origin of the malignant growth, whether it was first in the ileum or in the cœcum, but Dr. De Castel thinks that in the most cases which he dissected the growth started from the ileo-cœcal valve. By the narrowing of the ileum great masses of fecal matter are accumulated, and they are rejected by the stomach. The covering peritoneum is also mostly degenerated, and the pericœcal cellular tissue forms adhesions with the canal and the fossa. The diagnosis of the incipient cancer is very difficult, the symptoms being of a dyspeptic character at first, with general malaise, but later continued constipation and vomiting of fecal matter will show the character of the disease, and the presence of a tumor in the iliac region will make the diagnosis correct. The pain is but a secondary symptom, and it is often entirely wanting. The seat of the pain is either in the stomach (gastralgia), in the epigastrium, or around the navel. There are sometimes colic-like pains, which result from violent contractions of the intestine to overcome the narrowed canal. The vomiting is often observed in cases where the cœcum is not much involved. When the vomiting is fecal, it is tinged sometimes with blood resulting from hæmorrhage. The invasion of the peritoneum modifies the character of the disease, and there is either a tumor formed by the intestines adherent one to another, or there is a swelling of the abdomen which prevents the auscultation of the original tumor; in general the peritoneum is at last involved with the cancerous masses. If the glands are affected, the secondary cancer will attain a much larger size than that of the cœcum. The cancerous cachexia is often retarded. The course of the disease is short; from two months to one year. The treatment is confined to physics. Surgical treatment has never been tried in these cases, but it might become necessary in those cases which resemble strangulated hernia.—*Archives Général de Médecine.*

---

THAT dogs appreciate medical treatment is a fact that has been often attested. A story has lately been published illustrating this. One afternoon a little black-and-tan dog was found moaning piteously at the entrance of a hospital in East Concord street, Boston. The animal's apparent suffering attracted the attention of Dr. C. E. Hastings, one of the faculty, who made an examination, which revealed a bad compound fracture of the leg. The doctor set the bone, and gave the little sufferer such other treatment as the case required.—*Journal of Comparative Medicine and Surgery.*

